# Web-Based Tailored Intervention to Support Optimal Medication Adherence Among Kidney Transplant Recipients: Pilot Parallel-Group Randomized Controlled Trial

**DOI:** 10.2196/formative.9707

**Published:** 2018-07-19

**Authors:** José Côté, Marie-Chantal Fortin, Patricia Auger, Geneviève Rouleau, Sylvie Dubois, Nathalie Boudreau, Isabelle Vaillant, Élisabeth Gélinas-Lemay

**Affiliations:** ^1^ Research Chair in Innovative Nursing Practices Montreal, QC Canada; ^2^ Faculty of Nursing Université de Montréal Montreal, QC Canada; ^3^ Research Centre of the Centre Hospitalier de l’Université de Montréal Montreal, QC Canada; ^4^ Centre Hospitalier de l’Université de Montréal Montreal, QC Canada

**Keywords:** medication adherence, transplant recipient, self-management, nursing, Web-based tailored intervention, randomized controlled trial

## Abstract

**Background:**

Optimal immunosuppressive medication adherence is essential to graft survival. *Transplant-TAVIE* is a Web-based tailored intervention developed to promote this adherence.

**Objective:**

The objective of our study was to evaluate the *Transplant-TAVIE* intervention’s acceptability, feasibility, and preliminary efficacy.

**Methods:**

In a pilot, parallel-group, randomized controlled trial, we randomly assigned a convenience sample of 70 kidney transplant patients on immunosuppressive medication either to an experimental group (*Transplant-TAVIE*) or to a control group (existing websites). Kidney transplant recipients had to be older than 18 years, be taking immunosuppressant medication, and have access to the internet to participate in this study. *Transplant-TAVIE* was composed of three interactive Web-based sessions hosted by a virtual nurse. We documented user appreciation of and exposure to the intervention. Furthermore, we assessed medication adherence, medication self-efficacy, intake-related skills, and medication side effects at baseline and 3 and 6 months later. Analyses of variance were used to assess intergroup differences over time.

**Results:**

After baseline questionnaire completion, participants were randomly assigned either to *Transplant-TAVIE* (n=35) or to the websites (n=35) group. All participants had received their kidney graft <1 year to 32 years earlier (mean 6.8 years). Of the experimental group, 54% (19/35) completed the sessions of *Transplant-TAVIE*. Users found the intervention to be acceptable—33% were extremely satisfied (6/18), 39% were very satisfied (7/18), and 28% were satisfied (5/18). At baseline and over time, both experimental and control groups reported high medication adherence, high medication self-efficacy, and frequent use of skills related to medication intake. No intergroup differences emerged over time.

**Conclusions:**

The results of this study support the feasibility and acceptability of *Transplant-TAVIE*. It could constitute an accessible adjunct in support of existing specialized services.

## Introduction

### Background

Optimal immunosuppressive medication adherence is essential to graft survival [1,2]. However, lifelong daily intake of medication is a major challenge for kidney transplant patients. A meta-analysis revealed that across different types of transplantation, 19-25 per 100 patients per year were not adherent to immunosuppressant, and kidney recipients showed the highest rate of medication nonadherence of all (36 per 100 patients per year) [3].

In two separate systematic reviews of interventions aimed at enhancing medication adherence among kidney transplant patients, De Bleser et al (n=7) and Low et al (n=12) found interventions targeting multiple components—educational, behavioral, and affective—to be promising [4,5]. The evidence was only of a modest level, however, given the methodological limitations and small sample sizes of the studies reviewed. Similarly, in a scoping review, Oberlin et al concluded that no intervention was superior to another and proposed that transplant centers support medication adherence using multilevel strategies that include developing collaborative partnerships, stratifying the population, and employing multiple interventions [6]. In this regard, some researchers have suggested that technology could help improve and support medication adherence among kidney transplant recipients [4,7]. The use and added benefits of information and communication technologies (ICT) to support daily adherence in other patients with chronic conditions, such as cardiovascular diseases, asthma, or HIV, are well documented [8-10].

Against this background, we developed *Transplant-TAVIE,* a Web-based tailored nursing intervention, to empower kidney transplant recipients to manage their immunosuppressive drug treatment.

### Objective

The objective of our study was to evaluate the acceptability, feasibility, and preliminary efficacy of *Transplant-TAVIE* intended to support medication adherence among kidney transplant recipients.

## Methods

### Trial Design

We conducted a pilot, parallel-group, randomized controlled trial (RCT; 1:1 allocation ratio) to assess the acceptability and feasibility of the intervention. Adherence was the primary outcome measured. In addition, self-efficacy, skills, medication side effects, and self-perceived general state of health were secondary outcomes taken into consideration. There were three measurement times: baseline (T0) and 3 months (T3) and 6 months (T6) later. The study was approved by the Research Ethics Board of the Centre Hospitalier de l’Université de Montréal (CHUM). The RCT was reported according to the Consolidated Standards of Reporting Trials (CONSORT) statement guidelines for randomized pilot and feasibility trials [11]. We did not register the trial as recommended by the International Committee of Medical Journal Editors.

### Participants and Setting

The target population was composed of kidney transplant recipients followed up at the CHUM transplantation unit (Canada). The CHUM treats one of the largest cohorts of kidney transplant recipients in the province of Quebec (Canada). To participate in this study, patients had to be at least 18 years old, be on immunosuppressive medication, and have internet access. Anyone with an uncontrolled psychiatric or cognitive condition was excluded from the study.

At regular follow-up visits, potential participants were informed about the ongoing study by the unit receptionists who handed them a promotional flyer. The interested patients were invited to meet face-to-face with the research team in a room adjacent to the clinic, at which time the team went over a consent form to explain what the participation entailed. Patients who agreed to participate in the research signed the form. The baseline questionnaire was completed at the hospital, and follow-up questionnaires were completed by email or telephone at participants’ choice.

### Interventions

#### Experimental Group

*Transplant-TAVIE* was composed of three interactive Web-based sessions hosted by a virtual nurse, each 20- to 30-minute long. Over the course of sessions, users strengthened their sense of self-efficacy by developing and reinforcing self-management skills required for medication intake. The sessions aimed to help users incorporate the therapeutic regimen in their daily routine, cope with medication side effects, handle situations or circumstances that could interfere with medication intake, interact with health care professionals, and mobilize social support. The learning objectives included strengthening various capacities such as self-motivation and self-monitoring (session 1), problem solving and emotional control (session 2), and social interaction (session 3).

*Transplant-TAVIE* is modeled on the TAVIE (French acronym for Treatment, Virtual Nursing Assistance, and Education) concept and platform previously developed by Côté et al [12]. This intervention is informed by social learning theory and behavior change techniques [13]. Aside from delivering teaching, feedback, and positive reinforcement (verbal persuasion), the virtual nurse also refers to the experiences of other patients and holds them up as role models. The three sessions of *Transplant-TAVIE* are consecutive and follow a predefined sequence to ensure the gradual acquisition of knowledge and abilities (skills mastery).

**Figure 1:**
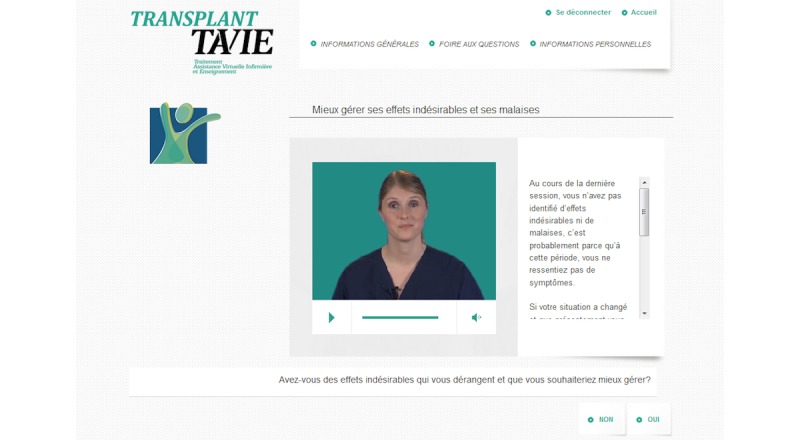
Screenshot of Transplant-TAVIE intervention.

*Transplant-TAVIE* was available only in French and contained 93 pages, 89 short videos and animated clips, and 58 PDF files (see Figure 1). Access to the intervention was unlimited in terms of intensity, frequency, and length of use between baseline and 3-month follow-up.

#### Control Group

Participants in the control group (CG) were invited to visit three predetermined conventional transplantation-related websites offering libraries of information. The websites belonged to three recognized organizations (ie, The Kidney Foundation of Canada, Canadian Transplant Association, and Transplant Companions) to ensure the reliability of content and quality of information. The choice of websites was validated by experts in the field of transplantation (MCF, NB, and IV).

The three main differences between CG and the experimental group (EG) lied in message tailoring, presence of a messenger (ie, the virtual nurse), and use of specific techniques or strategies based on theoretical methods. Accordingly, *Transplant-TAVIE* was a tailored intervention hosted by a virtual nurse that followed a decision tree, whereas the predetermined websites offered general information in written and graphic forms.

All participants had the interventions explained to them by a research assistant at the unit on the first study visit. A personalized reminder was sent to all participants by email or phone according to the participant preference 14 days after baseline to optimize participation in the interventions.

### Outcomes

#### Acceptability and Feasibility of Intervention

Intervention acceptability was measured on the Web-Based Nursing Intervention Acceptability Scale [14]. The 18-item scale covers nine dimensions: ease of navigation (2 items), ease of understanding (2 items), appreciation of nurse interaction and credibility of messenger (2 items), tailoring of information (2 items), individual pertinence (3 items), applicability (1 item), appreciation of user interface design (2 items), dosage (2 items), and general appreciation (2 items). Participants in the EG were handed the scale at baseline along with a prestamped envelope. They were asked to mail it to the research team after having completed the intervention. A personalized reminder was sent by the research assistant to participants who did not mail the questionnaire.

Intervention feasibility was assessed on the basis of intervention exposure. Participants in the EG were asked to sign up for the intervention by creating a user profile (ie, username and password). This one-time registration allowed data to be collected automatically on each user, including exposure to the intervention, pages most visited, time spent on pages, and PDF files most viewed.

We recorded the number of completed sessions for each participant. Intervention fidelity was determined by comparing the projected number of sessions (3) to the number of completed sessions. This was taken to reflect the feasibility.

#### Primary Outcome: Medication Adherence

We used two medication adherence measures. The *Immunosuppressant Therapy Adherence Instrument* is a 4-item scale with a potential score range of 0 (very poor adherence) to 12 (perfect adherence). The instrument has been found to be psychometrically sound: good validity (alpha, .81), strong intercorrelation between items (>0.84), and a single factor [15]. It is the first published scale to measure immunosuppressant therapy adherence.

We also assessed medication adherence using a visual analog scale from 0% to 100%.

#### Secondary Outcomes: Self-Efficacy, Skills, Medication Side Effects, and Self-Perceived General State of Health

The medication-taking self-efficacy was measured using 14 items rated on a 5-point scale ranging from 0% (“I cannot do it”) to 100% (“I am certain that I can”). The items were adapted from the *Long-Term Medication Behavior Self-Efficacy Scale* [16] and the barriers to adherence targeted by Chisholm et al among 222 graft recipients [17]. We carried out content validation and obtained a Cronbach alpha of .88 for this study.

A 24-item questionnaire rated on a 4-point Likert scale was developed for this study to assess medication intake–related skills such as motivation, self-observation, problem solving, emotion regulation, and social skills. Medication side effects were assessed with one question on whether signs or symptoms were present (yes or no) and how much discomfort was experienced (4-point scale ranging from “not at all” to “a lot”). We rated self-perceived general state of health on a visual analog scale.

Furthermore, we gathered sociodemographic data and information on transplant (ie, dialysis, wait time for transplantation, type of organ donor).

The preliminary effects regarding the primary outcome (medication adherence) and secondary outcomes (self-efficacy, skills, medication side effects, and self-perceived general state of health) were measured three times—at baseline and 3 and 6 months later. To promote participant engagement in the study, personalized reminder emails were sent out and direct calls were made. Participants were compensated for time spent completing the follow-up questionnaires; they received a Can $10 cheque after the second and third measurement time points.

### Sample Size

We planned a sample size of 70 participants (35 per group). No power calculations were performed. This sample size was justified by the fact that this was a pilot study [18].

### Randomization

As recommended in the CONSORT statement, participants were randomized after completion of the baseline assessment [19]. A permuted block randomization list (block size=10) was generated by computer. This method ensured a close balance between participants in each group at all times during the study. The allocation concealment mechanism consisted of copying the information about the randomization group (EG or CG) on a sheet and concealing it in consecutively numbered, opaque, and sealed envelopes. During data collection, one envelope was opened in front of each participant, thus, revealing the randomization group assigned to the participant.

### Blinding

Given the differences between the two interventions described in the consent form, participants were aware of the intervention they were randomized to. However, participants did not know which was the EG and which the CG. During data entry, the research team was blinded to group assignment (one database contained participant information and another only the collected data). All analyses were performed by an external statistician.

### Statistical Methods

Descriptive statistics such as frequency distribution and means with SDs were computed to describe the study population and intervention acceptability and feasibility. All patients were analyzed according to their randomized assignment. Analyses of variance (ANOVAs) were run to assess intergroup differences over time. Because of the small sample size, no missing data imputation was performed. Statistical significance was set at *P*=.05. All statistical analyses were performed using R freeware version 3.3.1 [20].

## Results

### Participant Flow

**Figure 2:**
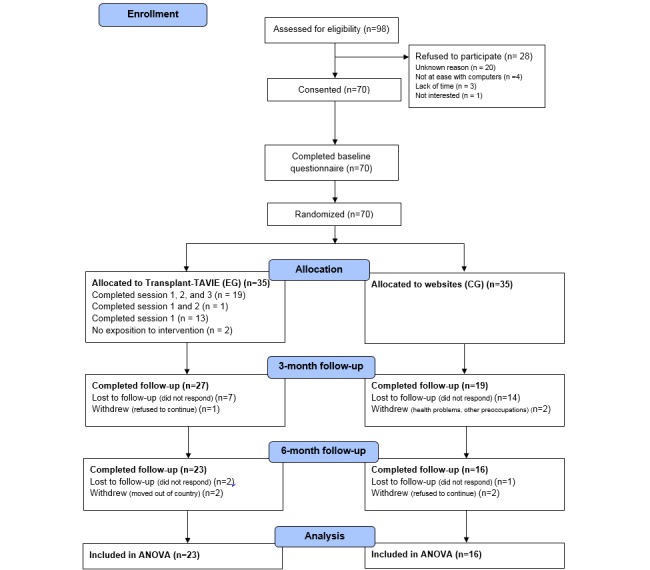
Participant flow diagram. ANOVA: analysis of variance.

The participant timeline, based on the CONSORT statement [11], is illustrated in Figure 2. Approximately 600 flyers were distributed by the transplantation unit staff to patients visiting the hospital for their usual follow-up. Overall, 98 patients responded and met face-to-face with a member of the research team. After being assessed for eligibility and being informed of what the research entailed, 70 patients consented to participate in the study for an acceptance rate of 71% (70/98). For the 28 patients who declined to participate, the principal reasons were lack of time, no access to a computer, and would think about it.

All 70 participants completed the baseline questionnaire and were randomized to either the EG (n=35) or the CG (n=35). The follow-up questionnaires were completed at 3 months postbaseline by 46 participants (EG: 27/35; CG: 19/35) and at 6 months postbaseline by 39 participants (EG: 23/35; CG: 16/35). More participants in the CG were considered lost to follow-up; there was a greater attrition in the CG than in the EG (19/35, 54% vs 12/35, 34%). In addition, participants lost to follow-up had a lower adherence mean score at baseline than those who completed both assessments (11.3 vs 11.7; *P*=.02). However, given that the maximum score on the adherence scale is 12, this difference was not clinically significant. In their study with 252 kidney transplant recipients, Weng et al defined nonadherent patients as those with a score of <9 on the Chisholm scale [21].

### Baseline Demographics and Clinical Characteristics

The detailed sociodemographic and clinical characteristics are presented in Table 1. Nearly two-thirds of the participants were male (EG: 24/35, 69%; CG: 22/35, 63%). The mean age was 54.03 years in the EG and 51.37 years in the CG (EG: range 36-75 years; CG: range 25-73 years). Nearly three-quarters of the participants had more than a high school education (EG: 25/32, 78%; CG: 23/32, 72%) and a little more than one-half worked full- or part–time (EG: 18/35, 51%; CG: 19/35, 54%). In addition, more than half lived with a partner (common law or married; EG: 28/34, 82%; CG: 25/34, 74%). Regarding clinical characteristics, most of the participants had been on dialysis before their transplantation (EG: 30/34, 88%; CG: 30/35, 86%). They had received their kidney graft <1 year to 32 years earlier (EG: mean 7.6 years, SD 7.3; CG: mean 6.1 years, SD 5.4).

### Intervention Acceptability

Of the participants randomized to receive *Transplant-TAVIE*, 51% (18/35) completed the acceptability questionnaire. All of them were generally satisfied with the virtual intervention (6/18, 33%, extremely satisfied; 7/18, 39%, very satisfied; and 5/18, 28%, satisfied).

All items regarding the ease of navigation, ease of understanding, appreciation of nurse interaction, and dosage were ranked positively. “Ease of navigation” referred to the ease with which the users surfed or moved about within the virtual intervention. Participants reported that the instructions were easy to follow: 12 of 17 said “totally easy” and 5 of 17 said “very easy” (1 case of missing data). They also reported that navigation within the virtual intervention was easy: 11 of 18 said “totally easy,” 6 of 18 said “very easy,” and 1 of 18 said “easy.” “Ease of understanding” referred to the users’ comprehension of the contents of the intervention. Participants reported that the language used by the virtual nurse was easy to understand (18/18, 100%) and that the content of the intervention was clear (17/18, 94%). Participants appreciated the interactions with the virtual nurse (18/18, 100%). Regarding the dosage, all (18/18, 100%) participants reported that the number of sessions was appropriate, and almost all (17/18, 94%) participants reported that the time allocated to each session was appropriate.

Regarding the appreciation of the user interface design, almost all (17/18, 94%) participants reported that the videos were interesting, and most (14/17, 82%) of them reported that the visual aspects were attractive. Most of the participants perceived the intervention to be useful (individual relevance): the intervention seemed appropriate to 83% (15/18), intervention helped with self-management of care in 72% (13/18), and the nurse proposed skills and strategies that met the needs of 89% (16/18) participants. All (18/18, 100%) of the completers felt that they were able to apply the tips and tricks recommended in the virtual intervention (applicability criteria); 83% (15/18) of the participants felt that they had access to a personalized consultation and 67% (12/18) felt that the messages in the virtual intervention were personally addressed to them. All (100%, 18/18) completers indicated that they would recommend it to other transplant recipients.

**Table 1:** Baseline sociodemographics and clinical characteristics.

Variable		*Transplant-TAVIE* (experimental group, n=35)		Websites (control group, n=35)	
**Sex, n (%)**					
	Male		24 (69)		22 (63)
	Female		11(31)		13 (37)
Age (years), mean (SD)			54.03 (9.75)		51.37 (11.99)
**Ethnic origin, n (%)**					
	Canadian		29 (83)		27 (77)
	Other		6 (17)		8 (23)
**Education (n=64), n (%)**					
	≤High school		7 (22)		9 (28)
	>High school		25 (78)		23 (72)
**Employment status, n (%)**					
	Employed		18 (51)		19 (54)
	Retired		11 (31)		6 (17)
	Unemployed		6 (17)		10 (29)
**Income, n (%)**					
	<Can $15,000		1 (3)		5 (14)
	Can $15,001-Can $30,000		3 (9)		6 (17)
	Can $30,001-Can $50,000		9 (26)		9 (26)
	Can $50,0001-Can $100,000		11 (31)		7 (20.0)
	Other		9 (26)		8 (23)
**Living situation, n (%)**					
	Alone		5 (14)		6 (17)
	With partner		18 (51)		22 (63)
	With family, friend, roommate		10 (29)		5 (14)
	Other		1 (3)		2 (6)
**Marital status (n=68), n (%)**					
	Single		4 (12)		7 (21)
	Married or living common law		28 (82)		25 (74)
	Divorced or widowed		2 (6)		2 (66)
**Kids (n=69), n (%)**					
	Yes		22 (65)		15 (43)
	No		12 (35)		20 (57)
**Dialysis before transplantation (n=69), n (%)**					
	Yes		30 (88)		30 (86)
	No		4 (12)		5 (14)
Years since transplantation, mean (SD)			7.6 (7.3)		6.1 (5.4)
Wait time before transplantation (in months), mean (SD)			35 (23)		36 (28)
**Type of kidney donor, n (%)**					
	Living		8 (23)		15 (43)
	Deceased		26 (74)		20 (57)

**Table 2:** Change in the adherence and secondary outcomes by the groups and over time (analysis of variance).

Variable		*Transplant-TAVIE* (experimental group), mean (SD)			Websites (control group), mean (SD)			Group × Time interaction, *F (P)*
		Baseline (n=35)	3-month follow-up (n=27)	6-month follow-up (n=23)	Baseline (n=35)	3-month follow-up (n=19)	6-month follow-up (n=16)	
Adherence score^a^		11.4 (1.0)	11.5 (0.8)	11.7 (0.6)	11.6 (0.7)	11.7 (0.7)	11.3 (2.0)	1.65 (.20)
Adherence visual scale^b^		97.1 (4.7)	96.8 (6.4)	98.7 (2.6)	98.3 (3.9)	98.7 (2.9)	98.9 (2.0)	0.45 (.64)
Self-efficacy^c^		1380.7 (60.7)	1397.7 (17.0)	1381.5 (37.9)	1391.4 (21.0)	1390.8 (17.1)	1393.8 (14.4)	1.02 (.37)
Skills^d^		81.3 (14.8)	79.8 (13.5)	78.6 (14.3)	79.0 (11.7)	75.3 (15.3)	77.4 (12.2)	0.94 (.39)
Degree bothered by side effects^e,f^		1.1 (1.4)	0.7 (1.0)	0.9 (1.2)	0.9 (1.3)	1.3 (1.2)	1.4 (1.2)	0.85 (.44)
Self-perceived state of health^g^		8.3 (1.1)	8.2 (1.2)	8.2 (1.4)	8.1 (2.0)	8.3 (1.7)	7.9 (2.2)	0.98 (.38)

^a^Possible score range: 0-12.

^b^Possible score range: 0-100.

^c^Possible score range: 0-1400.

^d^Possible score range: 0-96.

^e^Possible score range: 0-3.

^f^Among those who presented medication side effects.

^g^Possible score range: 0-10.

### Feasibility: Exposure to Intervention

In the EG, exposure to *Transplant-TAVIE* varied: 54% (19/35) completed all three sessions, 3% (1/35) completed only sessions 1 and 2, and 37% (13/35) completed only session 1. Furthermore, 6% (2/35) participants were not exposed to the intervention.

### Preliminary Efficacy of Intervention: Evolution of Adherence and Secondary Outcomes

The adherence scores were high in both the groups and remained stable over time (Table 2). At all three measurement times, both groups reported high self-perceived medication self-efficacy. Data revealed high self-confidence in the ability to take medication in different situations and frequent use of medication-taking skills. However, some participants were experiencing medication side effects and were slightly bothered by them. Most of the participants evaluated their general state of health as good. ANOVAs revealed no statistically significant differences between the groups or over time (Table 2).

## Discussion

### Principal Findings

The results of the study support the acceptability of the *Transplant-TAVIE* intervention. The EG participants generally appreciated the intervention in terms of the suitability of approach, convenience, ease of understanding, ease of use, and applicability of skills. However, some participants felt that the message from the virtual nurse could be more personalized to their needs. Given that the messages were prerecorded and presented following a pre-established algorithm, this remains a limitation of such an asynchronous intervention.

Regarding the intervention’s feasibility (ie, the extent of usage), 54% (19/35) participants completed all three sessions. This is congruent with the findings that emerged from the systematic review by Kelders et al to the effect that only an average of about 50% of participants adhered rigorously to the interventions of the sort [22]. The issue of engagement in Web-based and digital interventions is well documented in the literature [22-24]. In their systematic review of qualitative studies (n=19), O’Connor et al found that four factors affected patient engagement in digital health interventions: personal agency and motivation, priorities and values, contact with the intervention, and quality of the intervention [24]. The engagement in the health behavior is the starting point, and the technology remains a means to achieve this end.

In this study, the participants were already engaged in the behavior of taking medication and sought to achieve or maintain optimal adherence to their drug regimen. They had received their kidney graft many years earlier and had been taking medication since. The two adherence scores were high to begin with. Participants also reported high medication-taking self-efficacy and indicated frequently applying specific skills for the purpose of medication intake. In other words, our patients were already firmly engaged in the target behavior and they already used various strategies and skills in support of this behavior. Moreover, they were highly motivated and optimal medication intake was a priority for them.

The few ICT-based interventions offered in the field of nephrology have been proved highly acceptable to transplant recipients. For example, in a proof-of-concept trial, McGillicuddy et al found that a mobile phone-based remote health monitoring system developed to enhance medication adherence and blood pressure control enjoyed a high degree of acceptance among renal transplant recipients [25]. The added benefits of ICTs have also been documented for adolescents in terms of acceptability and feasibility [26]. Finally, in a review of mobile health resources in the field of solid organ transplants, Fleming et al documented the use of ICTs across the continuum from the pretransplant phase to the posttransplant phase [27]. However, the evidence amassed to date has been scant.

In a recent RCT conducted among kidney transplant recipients, Reese et al demonstrated that customized reminders, such as telephone calls, texting, and emails, significantly improved medication adherence compared with the usual treatment [28]. The main outcome, adherence, was measured using wireless pill-bottle openings. The researchers concluded that providing notifications and customized reminders showed promise as a measure to help patients improve adherence. As part of this pilot RCT, we aimed to determine the preliminary efficacy of the intervention by comparing the change in adherence scores between the EG and the CG. The results yielded no statistically significant intergroup difference in this regard.

The intervention studies conducted to date have focused on more traditional interventions and have demonstrated that these have a modest effect on medication adherence. In fact, in a meta-analysis of 8 studies involving 546 patients who received intervention through a pharmacist, intervention groups, or continuing education, Zhu et al found that adherence rates and adherence scores were significantly higher for the EG than for the CG [29].

In their systematic review of the literature, Low et al recommended that interventions target new transplant recipients and patients with medication adherence problems [5]. The question we must ask ourselves is who should a Web-based intervention such as *Transplant-TAVIE* target given the lower intensity of this type of intervention relative to support provided face-to-face? Should the target be people already engaged in the desired health behavior who are ready to engage in tech-based support? What about highly motivated individuals beginning treatment? In this regard, *Transplant-TAVIE,* like other interventions of the sort, is seen as adjunctive to the usual face-to-face care.

In addition, it is worth asking whether the intervention can appeal to individuals for whom medication adherence is a real problem, that is, whose suboptimal intake is related to a lack of motivation, shortage of resources, or limited capacity, or for whom the desired health behavior is not a priority. Given that individuals need to be motivated to engage in the health behavior in order to then engage in an eHealth intervention, does this sort of intervention serve the needs of people with real medication adherence problems? The fact that a Web-based intervention is accessible at all times in no way guarantees that it will be used.

According to Low et al, adherence enhancement efforts should focus on supportive, cost-effective, and multidimensional interventions [5]. Motivated people already under treatment or just beginning treatment are the ones most likely to benefit from ICT-based interventions adjunctive to usual or current care. People who have real difficulty taking medication, instead, would be better served by higher-intensity face-to-face interventions and more sophisticated intervention strategies better suited to reaching, attracting, and mobilizing this client group. Although a hybrid approach incorporating face-to-face and virtual interventions could be an interesting alternative, we recommend giving careful consideration to the opinions and needs of this patient group during the process of developing and implementing interventions [30].

### Strengths and Limitations

This study has some limitations. First, the attrition rate was high and more participants were lost to follow-up in the CG than in the EG (19/35, 54% vs 12/35, 34%). Second, as all the data collected were self-reported, social desirability and memory biases might have played a role in people’s responses. The results on the acceptability of the intervention reflect the point of view of half of the participants who returned their questionnaire. Finally, patients who accepted to participate in this study were not necessarily representative of the general transplant recipient population; they were highly educated and employed. Many of them were married or living common law; thus, most participants were not isolated. In future, researchers would do well to measure medication adherence more precisely and reliably by means of innovative tools and methods, such as remote wireless electronic monitoring of pill-bottle openings.

### Conclusions

Notwithstanding the limitations mentioned, we believe that the *Transplant-TAVIE*, a Web-based tailored nursing intervention, is acceptable and could constitute an accessible adjunct in support of existing specialized services. Further research is needed to determine more clearly the utility of this Web-based intervention for kidney transplant recipients beginning drug treatment. However, given that this treatment is life long, it is important to deploy interventions adapted to the different phases of the medication management continuum in order to support these patients more effectively.

## Appendix

Multimedia Appendix 1 CONSORT-EHEALTH checklist (V 1.6.1).

## Abbreviations

ANOVA: analysis of variance
CG: control group
CHUM: Centre Hospitalier de l’Université de Montréal
CONSORT: Consolidated Standards of Reporting Trials
EG: experimental group
ICT: information and communication technologies
