# Supplementary material for: Web-Based Tailored Intervention to Support Optimal Medication Adherence Among Kidney Transplant Recipients: Pilot Parallel-Group Randomized Controlled Trial
Source: JMIR Form Res. 2018 Jul 19;2(2):e14. doi: 10.2196/formative.9707 (PMC6334708; doi:10.2196/formative.9707)
Supplement: Multimedia Appendix 1 [file formative_v2i2e14_app1.pdf]

CONSORT-EHEALTH Checklist V1.6.2 Report	Manuscript Number	9707
**(based on CONSORT-EHEALTH V1.6), available at [http://tinyurl.com/consort-ehealth-v1-6].**		
**Date completed**		
3/13/2018 11:33:02		
**by**		
José Côté		
A web-based tailored intervention to support optimal medication adherence among kidney transplant recipients: a pilot parallel-group randomized controlled trial		
TITLE		
**1a-i) Identify the mode of delivery in the title**		
"A web-based tailored intervention"		
**1a-ii) Non-web-based components or important co-interventions in title**		
No. There is no non--web-based components.		
**1a-iii) Primary condition or target group in the title**		
"to support optimal medication adherence among kidney transplant recipients"		
ABSTRACT		
**1b-i) Key features/functionalities/components of the intervention and comparator in the METHODS section of the ABSTRACT**		
"In a pilot parallel-group randomized controlled trial, a convenience sample of 70 kidney transplant patients on immunosuppressive medication was randomly assigned either to an experimental group (Transplant-TAVIE) or to a control group (existing websites). Transplant-TAVIE was composed of three interactive web-based sessions hosted by a virtual nurse."		
**1b-ii) Level of human involvement in the METHODS section of the ABSTRACT**		
"A convenience sample of 70 kidney transplant patients on immunosuppressive medication was randomly assigned either to an experimental group (Transplant-TAVIE) or to a control group (existing websites).<br><br>Additionnal information (in method section of manuscript) : The allocation concealment mechanism consisted on copying the information about the randomization group (experimental or control) on a sheet and concealing it in consecutively numbered, opaque and sealed envelopes. During data collection, one envelope was open in front of each participant, thus revealing the randomization group assigned to each participant. "		
**1b-iii) Open vs. closed, web-based (self-assessment) vs. face-to-face assessments in the METHODS section of the ABSTRACT**		
**1b-iv) RESULTS section in abstract must contain use data**		
**1b-v) CONCLUSIONS/DISCUSSION in abstract for negative trials**		
INTRODUCTION		
**2a-i) Problem and the type of system/solution**		
"We developed Transplant-TAVIE, a web-based tailored nursing intervention, intended to empower kidney transplant recipients to manage their immunosuppressive drug treatment. Over the course of three interactive web-based sessions hosted by a virtual nurse, users strengthen their sense of self-efficacy by developing self-management skills required for medication intake. The sessions also aim to help users incorporate the therapeutic regimen in their daily routine, cope with drug side-effects, handle situations or circumstances that could interfere with medication intake, interact with health professionals, and mobilize social support."		
**2a-ii) Scientific background, rationale: What is known about the (type of) system**		

"Optimal immunosuppressive medication adherence is essential to graft survival [1, 2]. However, lifelong daily intake of medication is a major challenge for kidney transplant patients. A meta-analysis revealed that, across different types of transplantation, 19 to 25 per 100 patients per year were not adherent to immunosuppressant and kidney recipients showed the highest rate of medication non-adherence of all (36 per 100 patients per year) [3].<br><br>In two separate systematic reviews of interventions aimed at enhancing medication adherence among kidney transplant patients, De Bleser et al. (2009) (n=7) and Low et al. (2015) (n=12) found interventions targeting multiple components - educational, behavioural and affective - to be promising [4, 5]. The evidence was only of a modest level, however, given the methodological limitations and small sample sizes of the studies reviewed. Similarly, in a scoping review, Oberlin et al. (2016) concluded that no intervention was superior to another and proposed that transplant centers support medication adherence using multi-level strategies that include developing collaborative partnerships, stratifying the population and employing multiple interventions [6]. In this regard, some researchers have suggested that technology could help improve and support medication adherence among kidney transplant recipients [4, 7]. The use and added benefits of information and communication technologies (ICT) to support daily adherence in other patient suffering from chronic conditions, such as cardiovascular diseases, asthma or HIV, are well documented [8-10]."		
**Does your paper address CONSORT subitem 2b?**		
"We undertook a study to evaluate the acceptability, feasibility and preliminary efficacy of this intervention intended to support medication adherence among kidney transplant recipients."		
METHODS		
**3a) CONSORT: Description of trial design (such as parallel, factorial) including allocation ratio**		
"A pilot parallel-group randomized controlled trial (RCT) was conducted (1:1 allocation ratio) to assess the acceptability and feasibility of the intervention. Adherence was the primary outcome measured. Self-efficacy, skills, medication side-effects, and self-perceived general state of health were secondary outcomes taken into consideration. There were three measurement times: baseline (T0) and 3 months (T3) and 6 months (T6) later."		
**3b) CONSORT: Important changes to methods after trial commencement (such as eligibility criteria), with reasons**		
No. There were not important changes to methods after trial commencement.		
**3b-i) Bug fixes, Downtimes, Content Changes**		
**4a) CONSORT: Eligibility criteria for participants**		
"To participate in this study, patients had to: be at least 18 years old; be on immunosuppressive medication; and have Internet access. Anyone with an uncontrolled psychiatric or cognitive condition was excluded from the study."		
**4a-i) Computer / Internet literacy**		
**4a-ii) Open vs. closed, web-based vs. face-to-face assessments:**		
"At regular follow-up visits, potential participants were informed about the ongoing study by the unit receptionists who handed them a promotional flyer. Interested patients were invited to meet face to face with the research team in a room adjacent to the clinic, at which time the team went over a consent form to explain what participation entailed. Patients that agreed to participate in the research signed the form. The baseline questionnaire was completed at the hospital; follow-up questionnaires were completed by email or telephone at the participant's choice."		
**4a-iii) Information giving during recruitment**		
**4b) CONSORT: Settings and locations where the data were collected**		
"The target population was composed of kidney transplant recipients followed up at the Centre Hospitalier de l'Université de Montréal (CHUM) transplantation unit (Canada). The CHUM treats one of the largest cohorts of kidney transplant recipients in the province of Quebec (Canada)."		
**4b-i) Report if outcomes were (self-)assessed through online questionnaires**		
No. There was no online questionnaires in this trial.		
**4b-ii) Report how institutional affiliations are displayed**		
**5) CONSORT: Describe the interventions for each group with sufficient details to allow replication, including how and when they were actually administered**		

**5-i) Mention names, credential, affiliations of the developers, sponsors, and owners**		
**5-ii) Describe the history/development process**		
**5-iii) Revisions and updating**		
**5-iv) Quality assurance methods**		
**5-v) Ensure replicability by publishing the source code, and/or providing screenshots/screen-capture video, and/or providing flowcharts of the algorithms used**		
**5-vi) Digital preservation**		
**5-vii) Access**		
"The participants had the interventions explained to them by a research assistant at the unit on the first study visit."<br>Participants were given an explanatory sheet with how to access the platform (URL) and were invited to complete the intervention at home, at the moment of their choice.<br>"A personalized reminder was done - by email or phone according to the participant preference - 14 days after baseline to all participants in order to optimize participation in the interventions."<br>"Access to the intervention was unlimited in terms of intensity, frequency, and length of use between baseline and 3-month follow-up."<br>Access to the intervension were free.		
**5-viii) Mode of delivery, features/functionalities/components of the intervention and comparator, and the theoretical framework**		
"Experimental group (EG): Transplant-TAVIE is informed by social learning theory and behavior change techniques [12]. It is composed of three interactive web-based sessions, each 20–30 minutes long. The learning objectives included strengthening various capacities such as self-motivation and self-monitoring (session 1), problem solving and emotional control (session 2) and social interaction (session 3). The three sessions of Transplant-TAVIE are consecutive and follow a predefined sequence to maintain the gradual conveyance of abilities. Transplant-TAVIE was available only in French and contained 93 pages, 89 short video and animated clips and 58 PDF files (see Multimedia Appendix 1).<br><br>Control group (CG): Participants in the control group were invited to visit three predetermined conventional transplantation-related websites offering libraries of information. The websites belonged to three recognized organizations (i.e., The Kidney Foundation of Canada, Canadian Transplant Association and Transplant Companions) to ensure reliability of content and quality of information. The choice of websites was validated by experts in the field of transplantation (MCF, NB, IV, MJH)."		
**5-ix) Describe use parameters**		
**5-x) Clarify the level of human involvement**		
**5-xi) Report any prompts/reminders used**		
"A personalized reminder was done - by email or phone according to the participant preference - 14 days after baseline to all participants in order to optimize participation in the interventions."		
**5-xii) Describe any co-interventions (incl. training/support)**		
No. There was no co-intervention per se. Both interventions were adjunctive to usual face to face care provided by the clinic.		
**6a) CONSORT: Completely defined pre-specified primary and secondary outcome measures, including how and when they were assessed**		

"Acceptability and feasibility of intervention:<br>Intervention acceptability was measured on the Web-Based Nursing Intervention Acceptability Scale [14]. The 18-item scale covers nine dimensions: ease of navigation (2 items), ease of understanding (2 items), appreciation of nurse interaction/credibility of messenger (2 items), tailoring of information (2 items), individual pertinence (3 items), applicability (1 item), appreciation of user interface design (2 items), dosage (2 items), and general appreciation (2 items). Participants in the experimental group were handed the scale at baseline along with a pre-stamped envelope. They were asked to mail it to the research team after having completed the intervention. A personalized reminder was sent by the research assistant to participants who did not mail in the questionnaire.<br>Intervention feasibility was assessed on the basis of intervention exposure. Participants in the experimental group were invited to sign up for the intervention by creating a user profile (i.e. user name and password). This unique registration allows automated data collection for each user such as exposition to intervention, most visited pages, time spent on each pages, most viewed PDF files.<br>The number of completed sessions was recorded for each participant. Intervention fidelity was determined by comparing the projected number of session (3) to the number of completed sessions. This was taken to reflect feasibility.<br><br>Primary outcome - medication adherence:<br>Two medication adherence measures were used. The Immunosuppressant Therapy Adherence Instrument is a four-item scale with a potential score range of 0 (very poor adherence) to 12 (perfect adherence). The instrument has been found to be psychometrically sound: good validity ($\alpha = 0.81$), strong intercorrelation between items (> 0.84), and a single factor [15]. It is the first published scale to measure immunosuppressant therapy adherence. Medication adherence was also assessed with a visual analog scale from 0 to 100%.<br><br>Secondary outcomes - self-efficacy, skills, medication side-effects, self-perceived general state of health:<br>Medication-taking self-efficacy was measured with 14 items rated on a 5-point scale ranging from 0% ("I cannot do it") to 100% ("I am certain that I can"). The items were adapted from the Long-Term Medication Behavior Self-Efficacy Scale [16] and the barriers to adherence targeted by Chisholm et al. (2005) among 222 graft recipients [17]. Content validation was carried out and a Cronbach's alpha of 0.88 was obtained for this study.<br>A 24-item questionnaire rated on a 4-point Likert scale was developed for this study to assess medication-intake-related skills such as motivation, self-observation, problem solving, emotion regulation and social skills.<br>Medication side-effects were assessed with one question on whether signs or symptoms were present (yes or no) and how much discomfort was experienced (4-point scale ranging from "not at all" to "a lot").<br>Self-perceived general state of health was rated on a visual analog scale.<br><br>Sociodemographic data and information on transplant (i.e. dialysis, wait time for transplantation, type of organ donor) were gathered.<br><br>The primary and secondary outcomes (medication adherence, self-efficacy, skills, medication side-effects, self-perceived general state of health) were measured three times: at baseline and three and six months later. To encourage participants to persevere with the study, personalized reminder e-mails and phone calls were made. Participants were compensated for time spent for each follow-up questionnaire with a 10$ check after the second and third measurement time."		
**6a-i) Online questionnaires: describe if they were validated for online use and apply CHERRIES items to describe how the questionnaires were designed/deployed**		
**6a-ii) Describe whether and how "use" (including intensity of use/dosage) was defined/measured/monitored**		
**6a-iii) Describe whether, how, and when qualitative feedback from participants was obtained**		
**6b) CONSORT: Any changes to trial outcomes after the trial commenced, with reasons**		
"The target population was composed of kidney transplant recipients followed up at the Centre Hospitalier de l'Université de Montréal (CHUM) transplantation unit (Canada). The CHUM treats one of the largest cohorts of kidney transplant recipients in the province of Quebec (Canada)."		
**7a) CONSORT: How sample size was determined**		

**7a-i) Describe whether and how expected attrition was taken into account when calculating the sample size**		
**7b) CONSORT: When applicable, explanation of any interim analyses and stopping guidelines**		
"Acceptability and feasibility of intervention:<br>Intervention acceptability was measured on the Web-Based Nursing Intervention Acceptability Scale [14]. The 18-item scale covers nine dimensions: ease of navigation (2 items), ease of understanding (2 items), appreciation of nurse interaction/credibility of messenger (2 items), tailoring of information (2 items), individual pertinence (3 items), applicability (1 item), appreciation of user interface design (2 items), dosage (2 items), and general appreciation (2 items). Participants in the experimental group were handed the scale at baseline along with a pre-stamped envelope. They were asked to mail it to the research team after having completed the intervention. A personalized reminder was sent by the research assistant to participants who did not mail in the questionnaire.<br>Intervention feasibility was assessed on the basis of intervention exposure. Participants in the experimental group were invited to sign up for the intervention by creating a user profile (i.e. user name and password). This unique registration allows automated data collection for each user such as exposition to intervention, most visited pages, time spent on each pages, most viewed PDF files.<br>The number of completed sessions was recorded for each participant. Intervention fidelity was determined by comparing the projected number of session (3) to the number of completed sessions. This was taken to reflect feasibility.<br><br>Primary outcome - medication adherence:<br>Two medication adherence measures were used. The Immunosuppressant Therapy Adherence Instrument is a four-item scale with a potential score range of 0 (very poor adherence) to 12 (perfect adherence). The instrument has been found to be psychometrically sound: good validity ($\alpha = 0.81$), strong intercorrelation between items (> 0.84), and a single factor [15]. It is the first published scale to measure immunosuppressant therapy adherence. Medication adherence was also assessed with a visual analog scale from 0 to 100%.<br><br>Secondary outcomes - self-efficacy, skills, medication side-effects, self-perceived general state of health:<br>Medication-taking self-efficacy was measured with 14 items rated on a 5-point scale ranging from 0% ("I cannot do it") to 100% ("I am certain that I can"). The items were adapted from the Long-Term Medication Behavior Self-Efficacy Scale [16] and the barriers to adherence targeted by Chisholm et al. (2005) among 222 graft recipients [17]. Content validation was carried out and a Cronbach's alpha of 0.88 was obtained for this study.<br>A 24-item questionnaire rated on a 4-point Likert scale was developed for this study to assess medication-intake-related skills such as motivation, self-observation, problem solving, emotion regulation and social skills.<br>Medication side-effects were assessed with one question on whether signs or symptoms were present (yes or no) and how much discomfort was experienced (4-point scale ranging from "not at all" to "a lot").<br>Self-perceived general state of health was rated on a visual analog scale.<br><br>Sociodemographic data and information on transplant (i.e. dialysis, wait time for transplantation, type of organ donor) were gathered.<br><br>The primary and secondary outcomes (medication adherence, self-efficacy, skills, medication side-effects, self-perceived general state of health) were measured three times: at baseline and three and six months later. To encourage participants to persevere with the study, personalized reminder e-mails and phone calls were made. Participants were compensated for time spent for each follow-up questionnaire with a 10$ check after the second and third measurement time."		
**8a) CONSORT: Method used to generate the random allocation sequence**		
"A permuted block randomization list (block size = 10) was generated by computer."		
**8b) CONSORT: Type of randomisation; details of any restriction (such as blocking and block size)**		
"A permuted block randomization list (block size = 10) was generated by computer. This method ensured a close balance between participants in each group at all times during the study."		
**9) CONSORT: Mechanism used to implement the random allocation sequence (such as sequentially numbered containers), describing any steps taken to conceal the sequence until interventions were assigned**		

"The allocation concealment mechanism consisted on copying the information about the randomization group (experimental or control) on a sheet and concealing it in consecutively numbered, opaque and sealed envelopes. During data collection, one envelope was open in front of each participant, thus revealing the randomization group assigned to each participant."		
**10) CONSORT: Who generated the random allocation sequence, who enrolled participants, and who assigned participants to interventions**		
"The allocation concealment mechanism consisted on copying the information about the randomization group (experimental or control) on a sheet and concealing it in consecutively numbered, opaque and sealed envelopes."<br>The random allocation sequence was generated by a research assistant (GR).<br><br>"Interested patients were invited to meet face to face with the research team in a room adjacent to the clinic, at which time the team went over a consent form to explain what participation entailed. Patients that agreed to participate in the research signed the form."<br>The enrollment of participant was done by second research assistant (CD).<br><br>"During data collection, one envelope was open in front of each participant, thus revealing the randomization group assigned to each participant."<br>The assignement to interventions was done by a third research assistant (PA).		
**11a) CONSORT: Blinding - If done, who was blinded after assignment to interventions (for example, participants, care providers, those assessing outcomes) and how**		
**11a-i) Specify who was blinded, and who wasn't**		
"Given the differences between the two interventions described in the consent form, participants were aware of the intervention they were randomized to. However, participants did not know which was the experimental group and which the control group. During data entry, the research team was blinded to group assignment (one database contained participant information and another only the collected data). Analyses were performed by an external statistician."		
**11a-ii) Discuss e.g., whether participants knew which intervention was the "intervention of interest" and which one was the "comparator"**		
**11b) CONSORT: If relevant, description of the similarity of interventions**		
"The three main differences between the experimental and control conditions group and the experimental group had to do with tailoring the messenger (i.e., the virtual nurse), and use of specific techniques or strategies based on theoretical methods. Accordingly, Transplant-TAVIE was a tailored intervention hosted by a virtual nurse that followed a decision tree, whereas the predetermined websites offered general information in written and graphic form."		
**12a) CONSORT: Statistical methods used to compare groups for primary and secondary outcomes**		
"Descriptive statistics such as frequency distribution and means with standard deviations were computed to describe the study population and intervention acceptability and feasibility. T-tests and chi-square tests were used to compare the groups. All patients were analyzed according to their randomized assignment. ANOVAs were run to assess inter-group differences over time."		
**12a-i) Imputation techniques to deal with attrition / missing values**		
"Because of the small sample size, no missing data imputation was performed."		
**12b) CONSORT: Methods for additional analyses, such as subgroup analyses and adjusted analyses**		
No. No subgroup analyses and adjusted analyses were performed.		
RESULTS		
**13a) CONSORT: For each group, the numbers of participants who were randomly assigned, received intended treatment, and were analysed for the primary outcome**		
"After being assessed for eligibility and being informed of what the research entailed, 70 patients consented to participate in the study.<br>The 70 participants completed the baseline questionnaire and were randomized to either the experimental group (EG) (n=35) or the control group (CG) (n=35). The follow-up questionnaires were completed at 3 months post-baseline by 46 participants (27/35 EG, 19/35 CG) and at 6 months post-baseline by 39 participants (23/35 EG, 16/35 CG)."		
**13b) CONSORT: For each group, losses and exclusions after randomisation, together with reasons**		

"More participants in the control group were considered lost to follow-up: there was greater attrition in the CG than in the EG (54% (19/35) vs. 34%(12/35))."<br>These participants were considered lost to follow-up since they did not answer to the three reminders to complete the questionnaires. Thus, we do not have the reason.<br>There was no exclusions after randomisation.		
**13b-i) Attrition diagram**		
**14a) CONSORT: Dates defining the periods of recruitment and follow-up**		
Recruitment period: 2014-04-07 to 2014-10-23<br>Follow-up period: 2014-07-07 to 2015-04-29		
**14a-i) Indicate if critical “secular events” fell into the study period**		
**14b) CONSORT: Why the trial ended or was stopped (early)**		
The trial was stopped because projected sample size was reached.		
**15) CONSORT: A table showing baseline demographic and clinical characteristics for each group**		
See table 1 in manuscript.<br><br>"Detailed sociodemographic and clinical characteristics are presented in Table 1."		
**15-i) Report demographics associated with digital divide issues**		
" Nearly two-thirds of the participants were male (69% (24/35) in EG, 63% (22/35) in CG). Mean age was 54.03 years in the EG and 51.37 years in the CG (range: 36–75 in the EG, 25–73 in the CG). Nearly three-quarters had more than a high school education and a little more than one-half worked full or part time."<br><br>Ehealth literacy was not assessed.		
**16a) CONSORT: For each group, number of participants (denominator) included in each analysis and whether the analysis was by original assigned groups**		
**16-i) Report multiple “denominators” and provide definitions**		
Baseline sociodemographics and clinical characteristics descriptive analysis considered:<br>Transplant-TAVIE (EG): n=35<br>Websites (CG): n=35<br><br>Intervention acceptability descriptive analysis considered the Transplant-TAVIE (EG) users who completed the acceptability questionnaire: n = 18<br><br>Exposure to intervention descriptive analysis considered all participants in the Transplant-TAVIE (EG) group: n=35<br><br>ANOVA analysis to document preliminary efficacy of intervention considered:<br>Transplant-TAVIE (EG): Baseline, n=35; 3-month follow-up, n=27; 6-month follow-up, n=23<br>Websites (CG): Baseline, n=35; 3-month follow-up, n=19; 6-month follow-up, n=16<br><br>All analysis were by original assigned groups.		
**16-ii) Primary analysis should be intent-to-treat**		
**17a) CONSORT: For each primary and secondary outcome, results for each group, and the estimated effect size and its precision (such as 95% confidence interval)**		
See table 3 in manuscript.<br>Results for each group were presented.		

**17a-i) Presentation of process outcomes such as metrics of use and intensity of use**		
**17b) CONSORT: For binary outcomes, presentation of both absolute and relative effect sizes is recommended**		
Effect size was not presented.		
**18) CONSORT: Results of any other analyses performed, including subgroup analyses and adjusted analyses, distinguishing pre-specified from exploratory**		
No other analyses were performed.		
**18-i) Subgroup analysis of comparing only users**		
**19) CONSORT: All important harms or unintended effects in each group**		
No. There was no harms or uninteded effects in this study.		
**19-i) Include privacy breaches, technical problems**		
**19-ii) Include qualitative feedback from participants or observations from staff/researchers**		
DISCUSSION		
**20) CONSORT: Trial limitations, addressing sources of potential bias, imprecision, multiplicity of analyses**		
**20-i) Typical limitations in ehealth trials**		
"Our study is not without limitations. The attrition rate was high and more participants were lost to follow-up in the CG than in the EG (54% (19/35) vs. 34% (12/35)). Also, as all the data collected was self-reported, social desirability and memory biases might have played a role in people's responses. The results on the acceptability of the intervention reflect the point of view of half of the participants who returned their questionnaire. Also, the patients who accepted to participate in this study were not necessarily representative of the general transplant recipient population: they were highly educated and employed. Many of them were married or living common law, thus most were not isolated. In future, researchers would do well to measure medication adherence more precisely and reliably by means of innovative tools and methods, such as remote wireless electronic monitoring of pill-bottle openings."		
**21) CONSORT: Generalisability (external validity, applicability) of the trial findings**		
**21-i) Generalizability to other populations**		
**21-ii) Discuss if there were elements in the RCT that would be different in a routine application setting**		
**22) CONSORT: Interpretation consistent with results, balancing benefits and harms, and considering other relevant evidence**		
**22-i) Restate study questions and summarize the answers suggested by the data, starting with primary outcomes and process outcomes (use)**		
"The results of the study support the acceptability of the intervention. The EG participants generally appreciated the Transplant-TAVIE intervention in terms of suitability of approach, convenience, ease of understanding, ease of use and applicability of skills. However, some participants felt the intervention was not sufficiently tailored to their needs: they did not feel they received personalized messages from the virtual nurse. Given that the messages were pre-recorded and presented following a pre-established algorithm, this remains a limitation of such an asynchronous intervention."<br>"In our study, the participants were already engaged in the behavior of taking medication and sought to achieve or maintain optimal adherence to their drug regimen. They had received their kidney graft many years earlier and had been taking medication since. The two adherence scores were high to begin with. Participants also reported high medication-taking self-efficacy and indicated frequently applying specific skills for the purpose of medication intake."		
**22-ii) Highlight unanswered new questions, suggest future research**		
Other information		
**23) CONSORT: Registration number and name of trial registry**		
No. This study was not registered.		

**24) CONSORT: Where the full trial protocol can be accessed, if available**		
The full trial protocl cannot be accessed.		
**25) CONSORT: Sources of funding and other support (such as supply of drugs), role of funders**		
"The study is funded by the Kidney Foundation of Canada (2013-2015), Canadian Institutes of Health Research (CIHR, 2012-2013) and Research Chair in Innovative Nursing Practices. JC has received a clinical research bursary (Senior) from the Fonds de recherche du Québec - Santé (FRQS, 2013-2017) to support her research program on innovative virtual interventions that are intended for persons living with a chronic health problem. The TAVIE platform received financial support from the Réseau Sidami du FRSQ."		
**X26-i) Comment on ethics committee approval**		
"The study was approved by the Research Ethics Board of the Centre Hospitalier de l'Université de Montréal (CHUM)."		
**x26-ii) Outline informed consent procedures**		
**X26-iii) Safety and security procedures**		
**X27-i) State the relation of the study team towards the system being evaluated**		
